# Associations between prefrontal PI (16:0/20:4) lipid, *TNC* mRNA, and APOA1 protein in schizophrenia: A trans-omics analysis in post-mortem brain

**DOI:** 10.3389/fpsyt.2023.1145437

**Published:** 2023-04-18

**Authors:** Fumito Sano, Kenji Kikushima, Seico Benner, Lili Xu, Tomoaki Kahyo, Hidenori Yamasue, Mitsutoshi Setou

**Affiliations:** ^1^Department of Cellular and Molecular Anatomy, Hamamatsu University School of Medicine, Hamamatsu, Japan; ^2^Department of Psychiatry, Hamamatsu University School of Medicine, Hamamatsu, Japan; ^3^International Mass Imaging Center, Hamamatsu University School of Medicine, Hamamatsu, Japan; ^4^Department of Integrative Anatomy, Nagoya City University Graduate School of Medical Sciences, Nagoya, Japan; ^5^Department of Systems Molecular Anatomy, Institute for Medical Photonics Research, Preeminent Medical Photonics Education & Research Center, Hamamatsu University School of Medicine, Hamamatsu, Japan

**Keywords:** schizophrenia, postmortem brain, trans-omics, lipidomics, transcriptomics, proteomics

## Abstract

**Background:**

Though various mechanisms have been proposed for the pathophysiology of schizophrenia, the full extent of these mechanisms remains unclear, and little is known about the relationships among them. We carried out trans-omics analyses by comparing the results of the previously reported lipidomics, transcriptomics, and proteomics analyses; all of these studies used common post-mortem brain samples.

**Methods:**

We collected the data from three aforementioned omics studies on 6 common post-mortem samples (3 schizophrenia patients and 3 controls), and analyzed them as a whole group sample. Three correlation analyses were performed for each of the two of three omics studies in these samples. In order to discuss the strength of the correlations in a limited sample size, the *p*-values of each correlation coefficient were confirmed using the Student’s *t*-test. In addition, partial correlation analysis was also performed for some correlations, to verify the strength of the impact of each factor on the correlations.

**Results:**

The following three factors were strongly correlated with each other: the lipid level of phosphatidylinositol (PI) (16:0/20:4), the amount of *TNC* mRNA, and the quantitative signal intensity of APOA1 protein. PI (16:0/20:4) and *TNC* showed a positive correlation, while PI (16:0/20:4) and APOA1, and *TNC* and APOA1 showed negative correlations. All of these correlations reached at *p* < 0.01. PI (16:0/20:4) and *TNC* were decreased in the prefrontal cortex of schizophrenia samples, while APOA1 was increased. Partial correlation analyses among them suggested that PI (16:0/20:4) and *TNC* have no direct correlation, but their relationships are mediated by APOA1.

**Conclusion:**

The current results suggest that these three factors may provide new clues to elucidate the relationships among the candidate mechanisms of schizophrenia, and support the potential of trans-omics analyses as a new analytical method.

## 1. Introduction

Since the invention of omics research, we have been able to comprehensively detect and analyze the target molecular species of interest, and disease researches including psychiatric region have made further progress (1–3). However, there are still many diseases whose pathophysiology has not yet been fully elucidated. If the comprehensive analysis data obtained from the several omics studies can somehow be combined and analyzed more advanced, further research development could be expected, and fully understanding of pathophysiology of diseases could be achieved. Therefore, we would like to propose the possibility of a trans-omics analysis, that combines data from multiple omics studies performed on the same samples.

Previous studies using genome-wide association study (GWAS) or post-mortem brains of subjects with schizophrenia have reported genomics (4–7), transcriptomics (8), proteomics (9), and lipidomics (10) findings that characterize schizophrenia. For example, using GWAS, Psychiatric Genomics Consortium (PGC) identified 108 distinct loci associated with schizophrenia (11). Meta-analyses with new data have increased the susceptibility loci to 176 (12, 13). Development of transcriptome-wide association studies (TWAS) enables us to find genes whose expression is genetically correlated with schizophrenia (14, 15). In the proteomics study, alterations of aldolase C and glial fibrillary acidic protein of astrocytes (16) and myelin-associated proteins have been observed (16, 17). Our previous lipidomics study using liquid chromatography-electrospray ionization mass/mass spectrometry (LC-ESI/MS/MS) and imaging mass spectrometry (IMS) revealed that 16:0/20:4-phosphatidylinositol [PI (16:0/20:4)] levels specifically decrease in PFC of the brains from the patients with schizophrenia (18). Since recent research suggested that schizophrenia arise from an interaction between neurodevelopmental processes and environmental effects (19), it is essential to approach to understand the complex disease as schizophrenia from various aspects.

Previous studies using the post-mortem brain samples have suggested various mechanisms of schizophrenia pathogenesis, such as dopamine dysfunction (20), glutamate abnormalities (11), interneuron dysfunction (21), myelination defects (22), immune system disturbance (23), and oxidative stress (24). Though many factors have been reported in these different studies, little is known about the relationships of these pathways. An analysis in each omics layer represents just a slice of the whole complex biological system, which cannot indicate the interactions across multiple omics layers (25). Moreover, the various results presented by previous studies may simply reflect the heterogeneity of schizophrenia (26, 27). Since schizophrenia is a spectrum disorder characterized by many symptoms (28), and because their pathogenesis may vary from symptom to symptom, it is difficult to draw clear conclusions from the results of separate studies using different subjects.

In order to solve these problems, trans-omics analysis using the several omics data from the same brain samples is effective. Trans-omics analysis is attracting attention as a means to clarify relationships among multiple omics layers and to fill in the gaps between them (25). Since the usefulness of trans-omics analysis has been demonstrated in the various disease researches (29–31), applying the trans-omics approach to schizophrenia is similarly expected to elucidate mechanisms of schizophrenia that could not be revealed by each layer of omics, and lead to a discovery of the potential new therapeutic targets. Here, we conducted the trans-omics analysis using the data of our previous lipidomics (18), transcriptomics (32), and proteomics (33) studies, performed on the same post-mortem brain samples. With the publication of this analysis, we would like to contribute to the elucidation of the pathophysiology of schizophrenia, and to propose the potential of trans-omics researches.

## 2. Materials and methods

### 2.1. Human brain tissue samples

In the previous lipidomics, transcriptomics, and proteomics researches (18, 32, 33), BA10 in the prefrontal cortex of the post-mortem brain was used as a sample. Post-mortem brains of schizophrenia patients were from the Post-mortem Brain Bank of Fukushima for Psychiatric Research (Fukushima, Japan), and control group was from the Choju Medical Institute, Fukushimura Hospital (Toyohashi, Japan) (Table 1).

**Table 1:** Characteristics of subjects from whom postmortem brain samples were obtained.

Sample	Diagnosis	Age (years)	Sex	PMI (hours)	DOI (years)	Cause of death	Used in the lipidomics study^*1^	Used in the transcriptomics study^*2^	Used in the proteomics study^*3^
S01	SCZ	68	Female	15.0	40	Chronic renal failure	●	●	●
S02	SCZ	71	Male	16.5	48	Pneumonia	●	●	●
S03	SCZ	79	Female	17.0	60	Leukocytoclastic	●	●	●
									
C01	CON	85	Female	8.0	0	Pneumonia	●	●	●
C02	CON	89	Female	4.0	0	Heart failure	●	●	●
C03	CON	66	Male	42.0	0	Sudden death	●	●	●

^*1^Samples previously analyzed in the lipidomics study (18).

^*2^Samples previously analyzed in the transcriptomics study (32).

^*3^Samples previously analyzed in the proteomics study (33).

PMI: postmortem interval, the time that has elapsed since the person has died. DOI: duration of illness. SCZ: patients diagnosed with schizophrenia. CON: control subjects without schizophrenia.

Among those samples, 3 schizophrenia samples and 3 control samples were commonly used in these studies. There were no significant variations with the backgrounds of individuals between the 3 patients with schizophrenia and the 3 control subjects in age and gender. Five of the 6 samples had a post-mortem interval (PMI) of 17 h or less, and the remaining one had a PMI of 48 h. This research, including the use of post-mortem human brain tissue, was approved by the Ethics Committees of Hamamatsu University School of Medicine (the allowance number:14–179) and complied with the Declaration of Helsinki. All procedures were carried out with the informed written consent of the next of kin. All patients diagnosed with schizophrenia had fulfilled the diagnostic criteria established by the American Psychiatric Association (Diagnostic and Statistical Manual of Mental Disorders: DSM-IV).

### 2.2. Trans-omics analysis

The previous lipidomics (18), transcriptomics (32), and proteomics (33) researches identified 2 lipids, 7 differentially expressed genes (DEGs), and 14 proteins between schizophrenia and control samples, respectively. We collected the data of these factors in 6 common post-mortem samples (3 schizophrenia patients and 3 controls) from each omics research, and performed Dixon’s *Q* test with *Q*_99%_ to identify outliers in the data of collected factors. As a result of Q test, 2 proteins and 4 DEGs (eIF4G2, RUFY3, *COL1A2*, *COL6A2*, *PDGFRB*, *DDIT4*) were determined to contain outliers and these outliers were excluded from the analysis.

Then we performed correlation analyses with the 6 samples as a whole group sample. In order to perform correlation analyses among all factors, three different correlation analyses were performed for each of the two of three omics researches in these samples. To calculate the correlation coefficients, we used the signal intensities of the lipids, the mRNA amounts of the DEGs, and the quantitative signal intensities of the proteins obtained from previous omics researches. Correlation coefficients were calculated using Excel (Microsoft), and scatter plots were made by Kaleidagraph (Hulinks).

In order to discuss the strength of the correlations in a limited sample size, the *p*-values of each correlation coefficient were confirmed using the Student’s *t*-test. We obtained the *t*-values with the following equation.


$$t=\frac{\left|r\right|\sqrt{n-2}}{\sqrt{1-r^{2}}}$$

Since the sample size was undeniably small (6 samples, or 4 degrees of freedom), the α level of the *t*-test was strictly set at 0.01 with two-side, and only correlation coefficients that exceeded this level were focused on. In the *t*-distribution with 4 degrees of freedom, the *t*-value at the α level is *t* = 4.604. Substituting this into the above formula, we obtain *r* = 0.91719, which means that when the absolute value of the correlation coefficient (*r*) is 0.92 or higher, the *value of p* of the correlation coefficient is below 0.01.

For further analysis, we performed some partial correlation analyses to verify the strength of the impact of each factor on the correlations. A partial correlation analysis is controlled analysis for the intervention of confounding factors, so that we can distinguish a true correlation from spurious correlations. To calculate the partial correlation coefficient, the following equation was used.


$$r_{xy,z}=\frac{r_{xy}-r_{xz}r_{yz}}{\sqrt{\begin{array}{c} 1-r_{xz}^{2} \end{array}}\sqrt{1-r_{yz}^{2}}}$$

This ***r***_***xy,z***_ stands for “the partial correlation coefficient between x and y, after removing the effect of z.” Each of *x*, *y*, *z* in this equation can indicate any factor from each omics analysis: lipidomics, transcriptomics, and proteomics. Since it is difficult to verify the statistical significance of the partial correlation coefficients in this study, we simply compared the size of the coefficients after calculations.

## 3. Results

### 3.1. PI (16:0/20:4) strongly correlated with *TNC* gene

First, we evaluated the relationships between the lipidomics and transcriptomics factors. To evaluate the relationships, the correlation coefficients were analyzed using the 2 lipids and the 7 DEGs, those were identified in the previous researches (18, 32). PI (16:0/20:4) and phosphatidylserine(PS) (18:0/22:6) were identified from the lipidomics research. PI (16:0/20:4) had 6 (3 schizophrenias and 3 controls) samples in common with the other two omics researches as mentioned above, while PS(18:0/22:6) had only 4 (2 schizophrenias and 2 controls) samples in common, because the samples with PS(18:0/22:6) were partly different in the previous research. With 4 samples, or 2 degrees of freedom, ***r*** > 0.99 is required to exceed the α level of 0.01, so we could not identify any such correlation coefficients between PS(18:0/22:6) and other factors.

**Figure 1:**
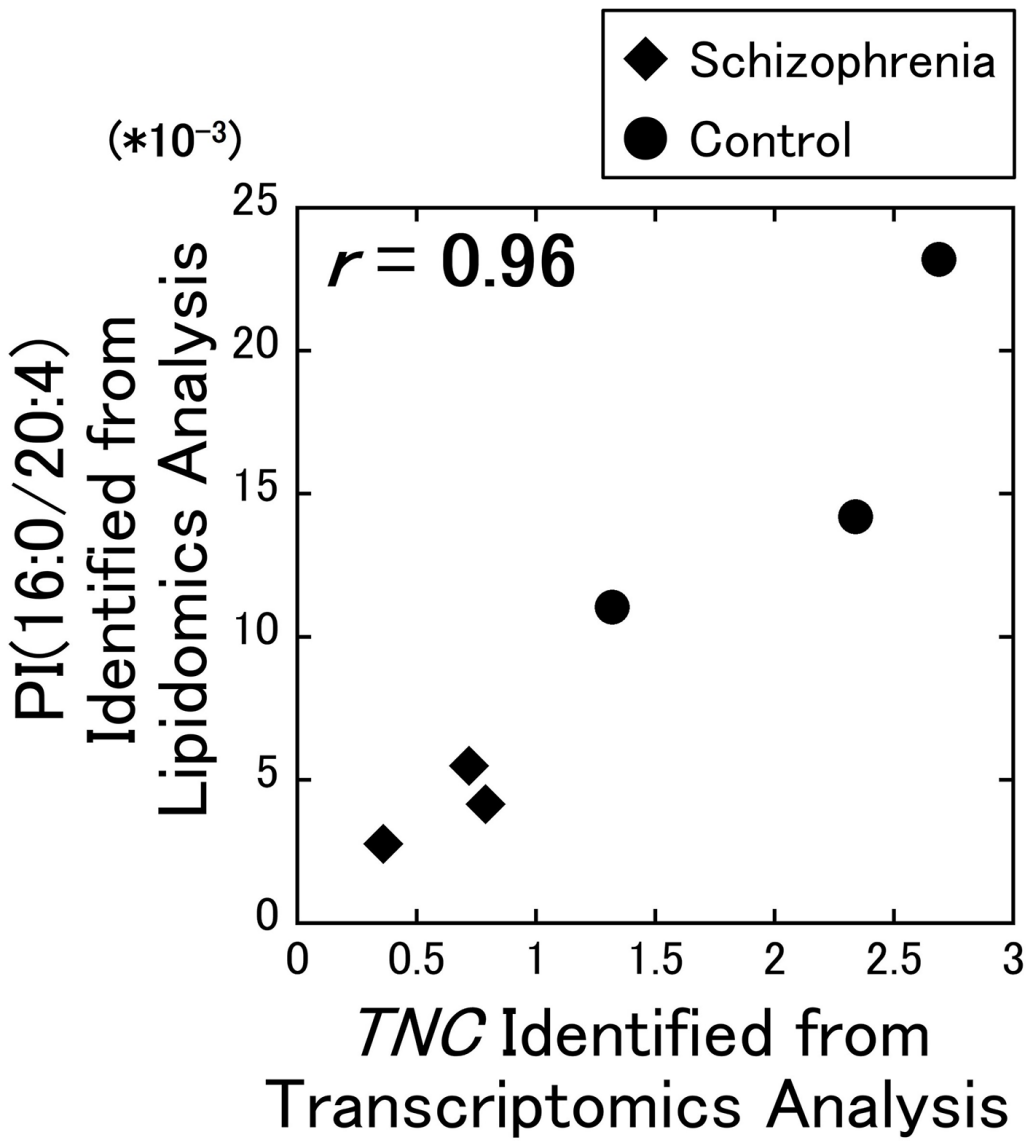
Relationship between the lipidomics and the transcriptomics factors. The signal intensity of the PI (16:0/20:4) were plotted against the RPKM of the *TNC* gene. All data including other lipids and DEGs are available on Supplementary Figure 1. Diamonds: samples from schizophrenia patients. Circles: samples from control subjects.

As a result of our analysis, we identified a strong positive correlation (***r*** = 0.96) between the PI (16:0/20:4) and the *TNC* gene (Figure 1), while no other correlation coefficients exceeded the alpha level of 0.01. Among the 7 DEGs, the *TNC* gene was the only gene that decreased with schizophrenia and showed a positive correlation with PI (16:0/20:4), while the other 6 DEGs (*COL1A2*, *COL6A2*, *DDIT4*, *FGF17*, *GNB3*, and *PDGFRB*) increased with schizophrenia and showed a negative correlation with PI (16:0 /20:4). The correlation coefficients between these 6 up-regulated DEGs and PI (16:0/20:4) remained in the range of −0.26 to −0.56.

In supplemental figures, the signal intensities of these lipids were plotted against fragments per kilobase of exon per million (FPKM) of the DEGs (Supplementary Figures 1A–N), and their correlation coefficients were determined and shown as a heatmap (Supplementary Figure 1O).

### 3.2. *TNC* gene strongly correlated with APOA1 protein and PBXIP1 protein

Second, we evaluated the relationships between the transcriptomics and proteomics factors. To evaluate the relationships, the correlation coefficients were analyzed using the 7 DEGs and 17 proteins, those were identified in the previous researches (32, 33).

**Figure 2:**
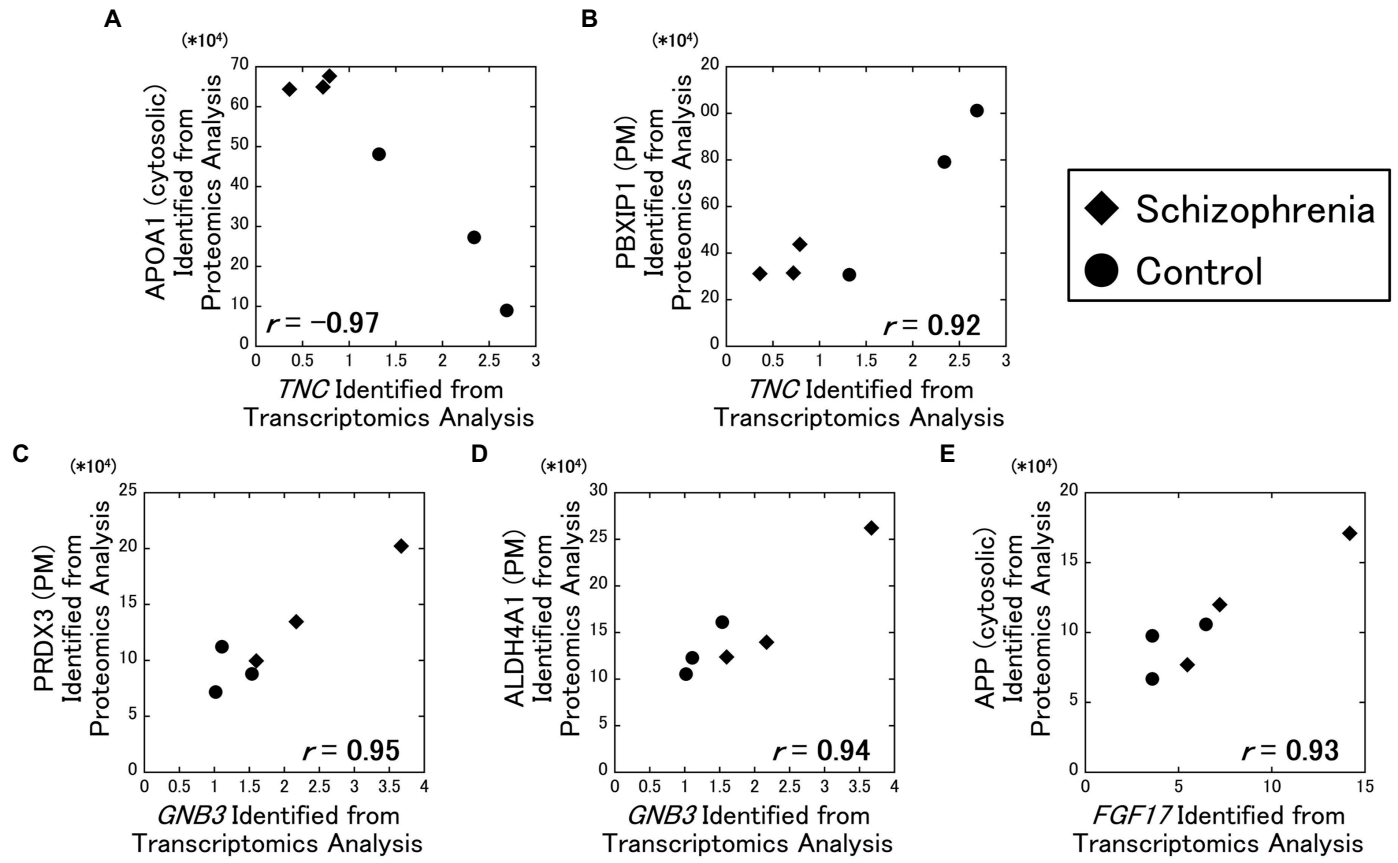
Relationships between the transcriptomics and the proteomics factors. The amounts of the identified proteins were plotted against RPKM of the DEGs. All data including other DEGs and proteins are available on Supplementary Figure 2. Diamonds: samples from schizophrenia patients. Circles: samples from control subjects.

As a result of our analysis, the *TNC* gene and the cytosolic APOA1 protein showed a strong negative correlation (***r*** = −0.97) (Figure 2A), while the *TNC* gene and the plasma membrane PBXIP1 protein showed a strong positive correlation (***r*** = 0.92) (Figure 2B). In addition, the *GNB3* gene and the plasma membrane PRDX3 protein showed a strong positive correlation (***r*** = 0.95) (Figure 2C), the *GNB3* gene and the plasma membrane ALDH4A1 protein showed a strong positive correlation (***r*** = 0.94) (Figure 2D), the *FGF17* gene and the cytosolic APP protein showed a strong positive correlation (***r*** = 0.93) (Figure 2E).

In supplemental figures, the quantitative signal intensities of the proteins were plotted against fragments per kilobase of exon per million (FPKM) of the DEGs (Supplementary Figures 2A–G), and their correlation coefficients were determined and shown as a heatmap (Supplementary Figure 2H).

For analyses including factors with outliers, analyses were conducted separately with the outliers excluded (Supplementary Figure 3). Their correlation coefficients did not exceed the α level of 0.01 when the outliers were excluded from the analyses.

### 3.3. PI (16:0/20:4) strongly correlated with APOA1 protein and PON2 protein

Third, we evaluated the relationships between the lipidomics and proteomics factors. To evaluate the relationships, the correlation coefficients were analyzed using the 2 lipids and 17 proteins, those were identified in the previous researches (18, 33).

**Figure 3:**
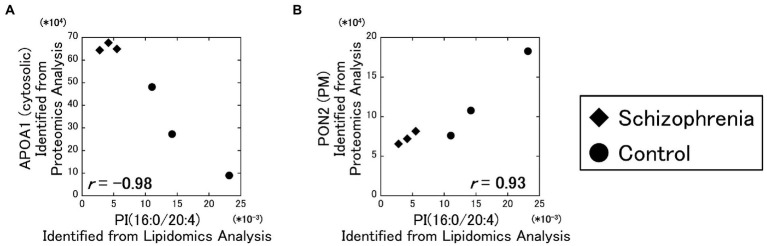
Relationships between the lipidomics and the proteomics factors. The signal intensity of the PI (16:0/20:4) were plotted against the amounts of the identified proteins. All data including other lipids and proteins are available on Supplementary Figure 3. Diamonds: samples from schizophrenia patients. Circles: samples from control subjects.

As a result of our analysis, the PI (16:0/20:4) and the cytosolic APOA1 protein showed a strong negative correlation (***r*** = −0.98) (Figure 3A), and the PI (16:0/20:4) and the plasma membrane PON2 protein showed a strong positive correlation (***r*** = 0.93) (Figure 3B). No other correlations exceeded the α level of 0.01.

In supplemental figures, the signal intensities of these lipids were plotted against the quantitative signal intensities of the proteins (Supplementary Figures 4A,B), and their correlation coefficients were determined and shown as a heatmap (Supplementary Figure 4C).

### 3.4. Partial correlation analyses between PI (16:0/20:4), *TNC* gene, and APOA1 protein

Through the above three correlation analyses, we found that the PI (16:0/20:4), the *TNC* gene, and the APOA1 protein are correlated with each other. Then, we performed partial correlation analyses on these three factors to determine the strength of their influence on each correlation.

**Figure 4:**
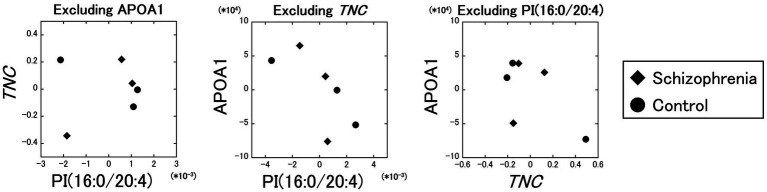
Partial correlation analyses between the PI (16:0/20:4), the *TNC* gene, and the APOA1 protein. The partial correlation between the PI (16:0/20:4) and the *TNC* gene, excluding the influence of the APOA1 protein, was comparatively poor (*r* = 0.11). The partial correlations between the PI (16:0/20:4) and the APOA1 protein excluding the influence of the *TNC* gene (*r* = −0.71), and between the *TNC* gene and the APOA1 protein excluding the influence of the PI (16:0/20:4) (*r* = −0.60) were comparatively strong. Diamonds: samples from schizophrenia patients. Circles: samples from control subjects.

Excluding the influence of the APOA1 protein, the partial correlation between the PI (16:0/20:4) and the *TNC* gene was comparatively poor (***r*** = 0.11). On the other hand, the partial correlations between the PI (16:0/20:4) and the APOA1 protein excluding the influence of the *TNC* gene (***r*** = −0.71), and between the *TNC* gene and the APOA1 protein excluding the influence of the PI (16:0/20:4) (***r*** = −0.60) were comparatively strong (Figure 4). These results showed that the partial correlation between the PI (16:0/20:4) and the *TNC* gene excluding the influence of the APOA1 protein was very weak (***r*** = 0.11) compared to the strength of the original correlation (***r*** = 0.96), while the partial correlations between the PI (16:0/20:4) and the APOA1 protein, and between the *TNC* gene and the APOA1 protein were not that weak (***r*** = −0.71 and ***r*** = −0.60) even when the influence of the *TNC* gene and the PI (16:0/20:4) was excluded, respectively.

### 3.5. Further analyses: Comparison with the dose of medication and age of death

Next, we compared each factor to the dose of antipsychotic medication and age of death (AoD). The doses of antipsychotic medication were standardized to chlorpromazine equivalents (CPeq), regardless of their type. Samples in the control group were analyzed as 0 mg/day of CPeq.

As a result of our analysis, the PI (16:0/20:4) and CPeq (***r*** = −0.82), the PI (16:0/20:4) and AoD (***r*** = 0.65), the *TNC* gene and CPeq (***r*** = −0.82), the *TNC* gene and AoD (***r*** = 0.76), the APOA1 protein and CPeq (***r*** = 0.79), the APOA1 protein and AoD (***r*** = −0.70), showed comparatively strong correlations, though the α level of 0.01 were not exceeded. Then we performed partial correlation analyses to evaluate the relationship between each factor and Cpeq, excluding the effect of AoD. The partial correlations between the PI (16:0/20:4) and CPeq (***r*** = −0.74), between the *TNC* gene and CPeq (***r*** = −0.76), and between the APOA1 protein and CPeq (***r*** = 0.69), were all relatively strong.

To confirm that the correlations between each pair of the PI (16:0/20:4), the *TNC* gene, and the APOA1 protein were not spurious correlations that merely reflected the correlations with CPeq and AoD, we next performed partial correlation analyses that excluded the effects of CPeq and AoD. The partial correlations between the PI (16:0/20:4) and the *TNC* gene excluding the influence of CPeq (***r*** = 0.87), between the *TNC* gene and the APOA1 protein excluding the influence of the CPeq (***r*** = −0.93), between the APOA1 protein and the PI (16:0/20:4) excluding the influence of the CPeq (***r*** = −0.94), were all strong. Also, the partial correlations between the PI (16:0/20:4) and the *TNC* gene excluding the influence of AoD (***r*** = 0.94), between the *TNC* gene and the APOA1 protein excluding the influence of the AoD (***r*** = −0.95), between the APOA1 protein and the PI (16:0/20:4) excluding the influence of the AoD (***r*** = −0.97), were all strong too.

## 4. Discussion

In this study, we combined and analyzed the data from three previous omics studies: lipidomics, transcriptomics, and proteomics (18, 32, 33). These three studies had six common samples, which allowing each omics factors, and found several strong correlations in our analyses.

In this study, correlation coefficients were calculated with the factors that had been significantly different in previous omics studies, so it would seem that correlations would inevitably appear as well, but the results were different from expectations. As shown in the results of our study, only several pairs showed correlations exceeding the α level of 0.01, and most of the combinations did not show strong correlations. We make the following assumption about these results. The significant difference found in the previous study leads to the result that the SCZ and Ctrl groups are distributed apart in the scatterplot when the two factors are set on the X and Y axes, as in our study, but this does not necessarily mean that they are aligned in a straight line and show a correlation. The SCZ and Ctrl groups, which are distributed apart, are more likely to appear on the scatterplot as two separate lines or show unconnected distributions than to be grouped together as a single line in their entirety.

Samples in the SCZ group were provided by a brain bank in Fukushima Prefecture, and samples in the Ctrl group were provided by a brain bank in Aichi Prefecture. Therefore, samples from different institutions were compared. However, both institutions are in Japan, and it is thought that there is no difference in the race or living environment of the sample donors, and that the difference in institutions has little influence on the samples and the analysis. The same samples were also used in the three previous omics studies (18, 32, 33), but the differences in facilities was not a major issue in these previous studies either, and it can be assumed that the influence of facility differences on the sample and analysis was determined to be small.

In the analysis of the lipidomics data and the transcriptomics data, we identified the strong correlation between the PI (16:0/20:4) and the *TNC* gene (***r*** = 0.96). The results of the partial correlation analysis suggested that the correlation of these two factors might be mediated by the APOA1 protein, and their direct correlation was seemed to be poor (***r*** = 0.11).

PI (16:0/20:4) is a PI containing palmitic and arachidonic acids as fatty acid chains and is found on the cytosolic side of the cell membrane. *TNC* gene encodes the Tenascin C protein, which is a member of the tenascin family, comprised of glycoproteins, and highly expressed in the extracellular matrix. Tenascin C has interactions with certain cell surface receptors such as epidermal growth factor receptor, integrins, Toll-like receptor 4, and Wnt pathway. These interactions lead to changes in gene transcription, resulting in changes of expression of proteins involved in proliferation, migration, adhesion, cell survival and apoptosis, differentiation, synaptic activity and immune response. Because of its diverse action sites and functions, it is difficult to understand all aspects of the functions of Tenascin C. For example, studies in animal models of various diseases have reported both pro-inflammatory and anti-inflammatory effects of Tenascin C (34, 35). In addition, almost no studies have discussed the relationship between Tenascin C and psychiatric disorders, making it difficult to discuss the significance of reduced Tenascin C in the prefrontal cortex (BA10) of schizophrenia patients. If Tenascin C is reduced in the dentate gyrus of the hippocampus and the subventricular zone around lateral ventricles, as well as in BA10, it may be possible that reduced Tenascin C is associated with reduced neurogenesis.

In the analysis of the transcriptomics data and the proteomics data, we identified the strong correlations between the *TNC* gene and the APOA1 protein (***r*** = −0.97), and between the *TNC* gene and the PBXIP1 protein (***r*** = 0.92). The results of the partial correlation analysis suggested that the correlation of the *TNC* gene and the APOA1 protein was comparatively strong (***r*** = −0.60) under excluding the influence of the PI (16:0/20:4). This result suggests that the *TNC* gene and the APOA1 protein may have some direct relationship.

APOA1 protein is one of the major components of high-density lipoprotein (HDL) and is involved in lipid metabolism. The relationship between HDL and schizophrenia has already been reported several times. For example, serum low HDL level has been reported to be associated with aggression in female patients with schizophrenia (36), and elevated serum HDL during the first year of drug treatment for schizophrenia has been reported to be associated with less negative symptoms (37). In addition, APOA1 in HDL has a variety of functions, including binding to the cell surface scavenger receptor class B type I (SR-BI) to activate Src and mediate the PI3K, Akt, and MAPK/ERK pathways. Consequently, APOA1 activates NO production in vascular endothelial cells, activates HIF-1α and VEGF under hypoxia, and suppresses HIF-1α and VEGF under inflammation (38, 39). APOA1 has diverse functions beyond multiple pathways like Tenascin C, however, there are no many researches written on the relationship between Tenascin C and either APOA1 or HDL, especially in psychiatric region. The negative correlation between *TNC* gene and APOA1 protein must have some implications, but at this point it is unclear.

PBXIP1 protein is a suppressive regulator of pre-B-cell leukemia transcription factors, and it also interacts with estrogen receptor α and β (ERα, ERβ). Estradiol is used as adjunctive treatment in female schizophrenia patients, and has been reported to improve their positive and negative symptoms (40, 41). It is also suspected to be the cause of the gender differences of schizophrenia and has attracted attention for its involvement in the pathophysiology of schizophrenia. Besides, another research showed that the Estradiol receptors ERα and ERβ improve hippocampal memory function by activating the ERK pathway, while GPER, a G protein-coupled estrogen receptor, improves hippocampal memory function by activating the JNK pathway (42). *TNC* gene PBXIP1 protein were both decreased in schizophrenia in this analysis, and it might be possible that both were decreased in association with reduced hippocampal function and neurogenesis.

In the analysis of the lipidomics data and the proteomics data, we identified the strong correlations between the PI (16:0/20:4) and the APOA1 protein (***r*** = −0.98), and between the PI (16:0/20:4) and the PON2 protein (***r*** = 0.93). The results of the partial correlation analysis suggested that the correlation of the PI (16:0/20:4) and the APOA1 protein was comparatively strong (***r*** = −0.71) under excluding the influence of the *TNC* gene. This result suggests that the PI (16:0/20:4) and the APOA1 protein may have some direct relationship.

PON2 protein is expressed throughout the body, including the brain, and has antioxidant properties. In the brain, PON2 is particularly strongly expressed in the dopaminergic nervous system, such as the striatum, the nucleus accumbens, and the substantia nigra (43, 44). PON2 prevents neurodegeneration by providing a protection against oxidative stress-mediated neurotoxicity. At the plasma membrane, PON2 is a transmembrane protein with its enzymatic domain facing the extracellular compartment, while it is localized at the perinuclear region, the endoplasmic reticulum, and the mitochondria, in the cytoplasm. Plasma membrane PON2 protein serves to prevent peroxidation of membrane components, primarily lipids. Based on this, it appears that the correlation between PI (16:0/20:4) and PON2 protein is due to the accelerated peroxidation of plasma membrane lipids caused by the decrease of PON2, which leads to a decrease of PI (16:0/20:4) in the plasma membrane. In addition, “antioxidant properties” and “localization to the dopaminergic nervous system” of PON2 protein mean that it may be related to both the oxidative stress hypothesis and the dopamine hypothesis, which are the major pathophysiological hypotheses of schizophrenia, and may attract attention to PON2 protein as a new research/therapeutic target.

In the partial correlation analyses, we identified that the partial correlation coefficients between each pair of the PI (16:0/20:4), the *TNC* gene, and the APOA1 protein, were still strong under excluding the influence of AoD and CPeq (0.87 ≤ | ***r*** | ≤ 0.97). These partial correlation coefficients indicated that AoD and CPeq had only little influence on the correlations between each pair of the PI (16:0/20:4), the *TNC* gene, and the APOA1 protein. In addition, the partial correlation coefficient between the PI (16:0/20:4) and the *TNC* gene excluding the influence of the APOA1 protein, was poorer than those excluding the influence of AoD and CPeq. The results for the other combinations were same, of course. These analyses indicated that the correlations between each pair of the PI (16:0/20:4), the *TNC* gene, and the APOA1 protein, were more strongly influenced by the rest one of these factors than by AoD and CPeq.

To summarize so far, we identified the certain correlations between the factors which extracted from different omics studies, using same samples of Schizophrenia post-mortem brains. Particularly, the correlations between the PI (16:0/20:4) and the APOA1 protein, and between the *TNC* gene and the APOA1 protein were strong and robust. However, while we have demonstrated these correlations, we have not been able to determine what these correlations mean and how they relate to the pathophysiology of schizophrenia. But that is not important this time. It is a great achievement that we were able to identify the aforementioned interesting correlations and propose new targets for future research, from a small sample of only six cases. This kind of approach to trans-omics analysis has the potential to produce certain results even with a small number of samples as in this case. It is of course useful in regions where researches have already progressed, but it can also be useful in regions where researches are difficult to progress due to a small number of cases or reports. We would like to propose the possibility of trans-omics analysis as a new method to overcome the problem of “sample size,” which always stands as a practical limitation against the verification of scientific significance.

## 5. Conclusion

We performed the trans-omics analyses, using the data extracted from our previous omics researches: lipidomics, transcriptomics, and proteomics. These previous researches commonly used 6 same post-mortem brain samples of Schizophrenia patients and controls. We identified the certain strong correlations, and particularly the correlations between each pair of the PI (16:0/20:4), the *TNC* gene, and the APOA1 protein seemed to be strong. We would like to propose the possibility of trans-omics analysis as a new method to overcome the problem of sample size and explore new research and therapeutic targets.

## Data availability statement

The original contributions presented in the study are included in the article/Supplementary material, further inquiries can be directed to the corresponding author.

## Ethics statement

The studies involving human participants were reviewed and approved by Ethics Committees of Hamamatsu University School of Medicine. Written informed consent for participation was not required for this study in accordance with the national legislation and the institutional requirements.

## Author contributions

FS and KK had contribution in performing the analyses and writing the manuscript discussing with SB, LX, TK, HY, and MS. MS supervised the entire project. All authors contributed to the article and approved the submitted version.

## Funding

This study was funded by the Chronology of the Ministry of Education, Culture, Sports, Science and Technology (MEXT) Project: JPMXS0410300220, and the Japan Agency for Medical Research and Development (AMED): JP20gm0910004. AMED had no role in the study design, data collection, data analyses and interpretation, and the decision to submit the report for publication.

## Conflict of interest

The authors declare that the research was conducted in the absence of any commercial or financial relationships that could be construed as a potential conflict of interest.

The handling editor MH declared a past co-authorship with the author MS.

## Publisher’s note

All claims expressed in this article are solely those of the authors and do not necessarily represent those of their affiliated organizations, or those of the publisher, the editors and the reviewers. Any product that may be evaluated in this article, or claim that may be made by its manufacturer, is not guaranteed or endorsed by the publisher.
