# Supplementary material for: Associations between prefrontal PI (16:0/20:4) lipid, TNC mRNA, and APOA1 protein in schizophrenia: A trans-omics analysis in post-mortem brain
Source: Front Psychiatry. 2023 Apr 18;14:1145437. doi: 10.3389/fpsyt.2023.1145437 (PMC10151580; doi:10.3389/fpsyt.2023.1145437)
Supplement: Supplementary file 2 [file Data_Sheet_1.docx]

**Supplementary Figures**


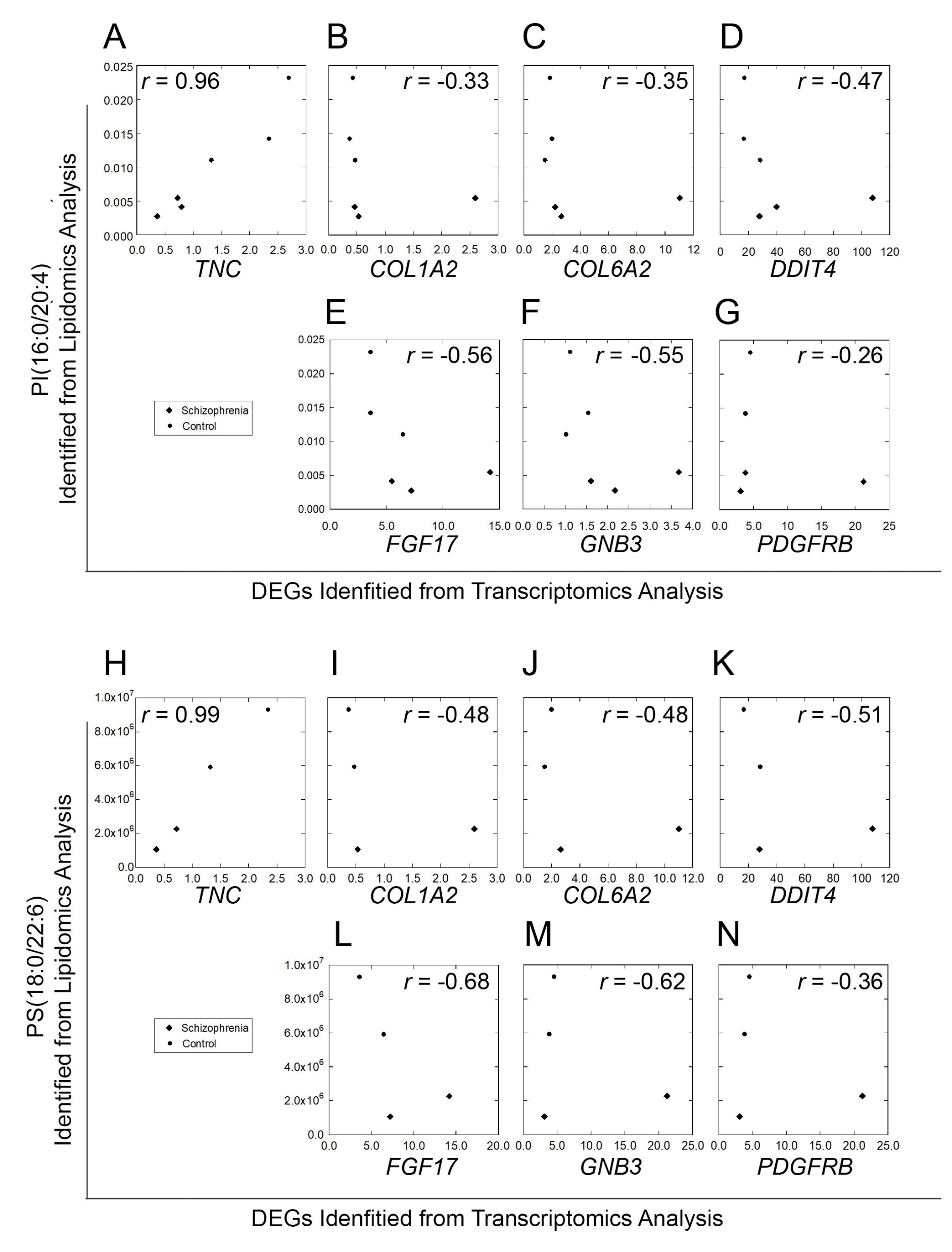


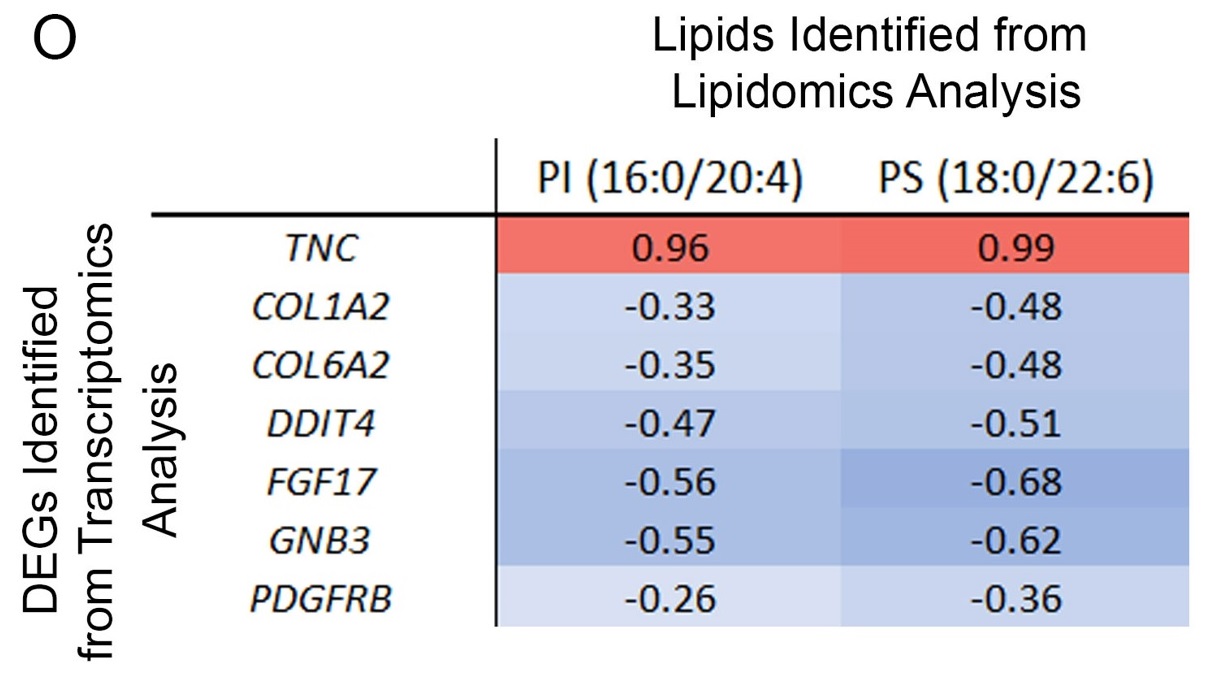


**Supplementary Figure 1.** Relationships between the lipidomics and the transcriptomics factors

(**A-N**) The signal intensity of the lipids, PI(16:0/20:4) and PS(18:0/22:6) were plotted against the RPKM of the 7 DEGs. Diamonds: samples from schizophrenia patients. Circles: samples from control subjects.

(**O**) Heatmap of the correlation coefficients between the factors identified from the lipidomics and the transcriptomics analyses. The red color suggests a positive correlation, while the blue color suggests a negative correlation.

**A**

**
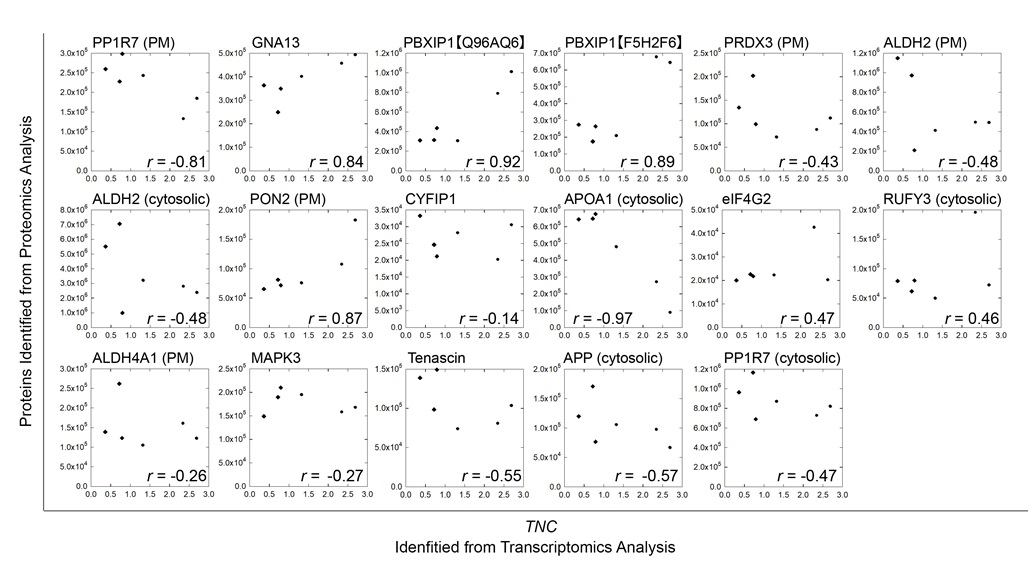
**

**B**

**
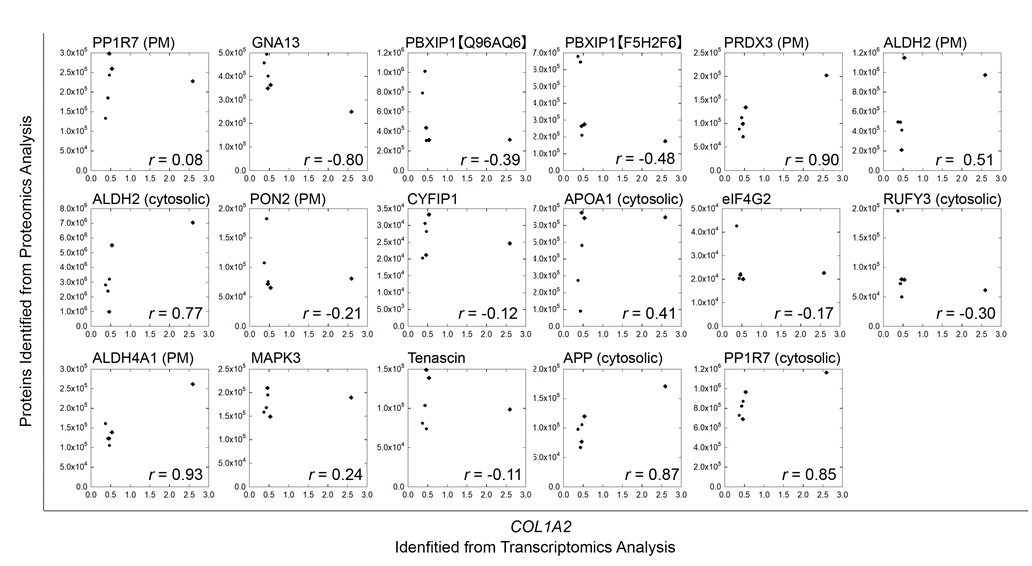
**

**C**

**
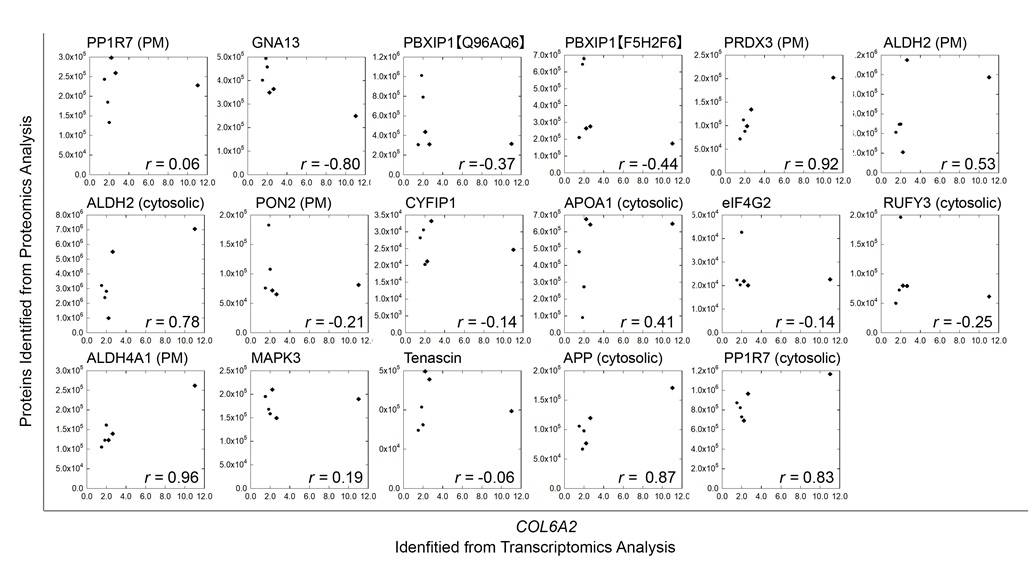
**

**D**

**
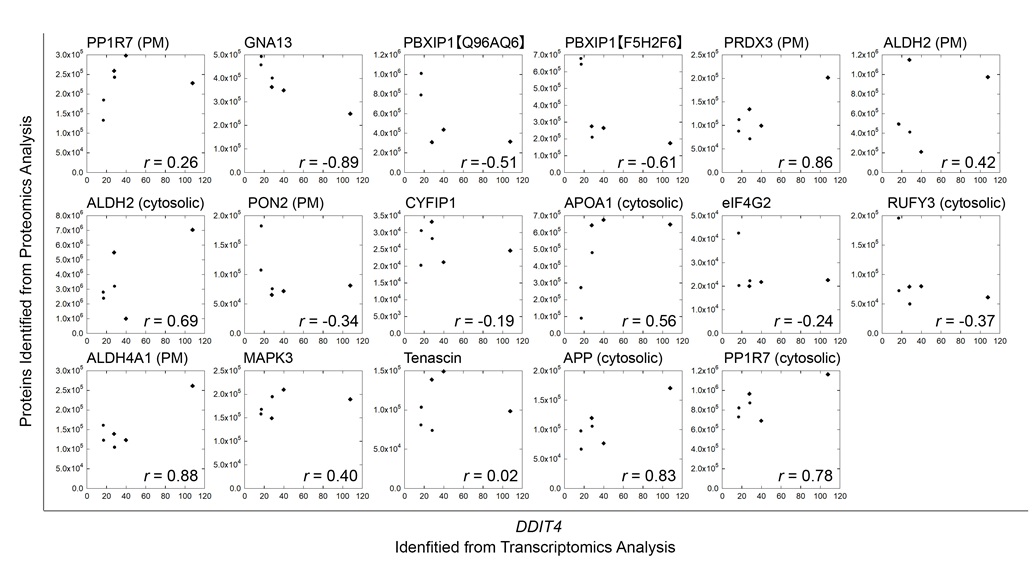
**

**E**

**
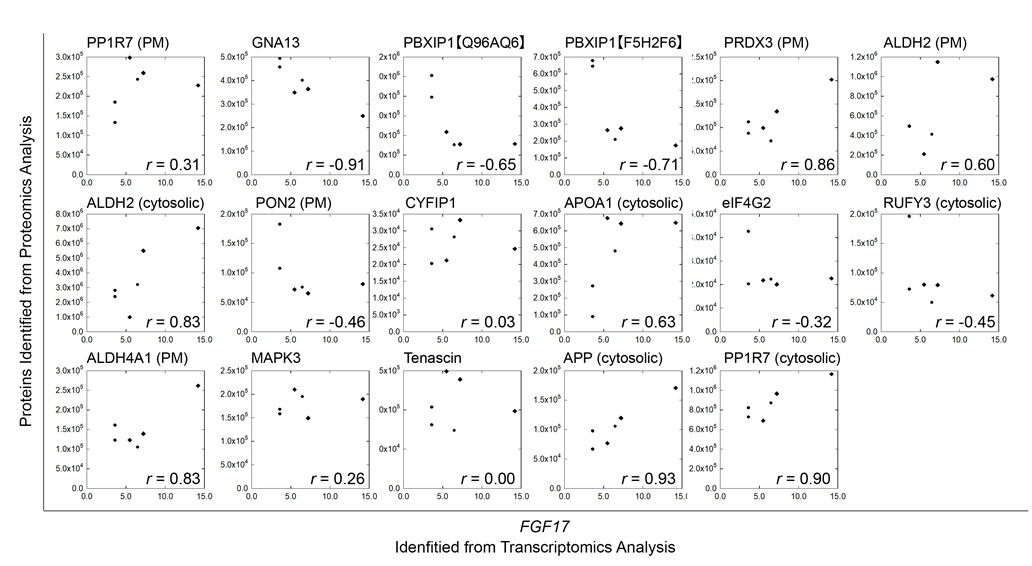
**

**F**

**
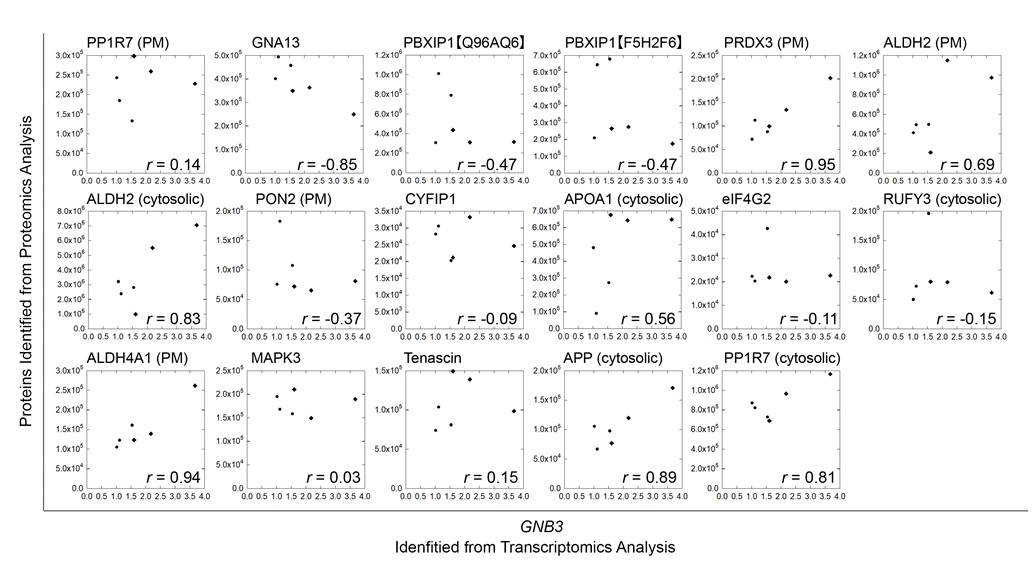
**

**G**

**
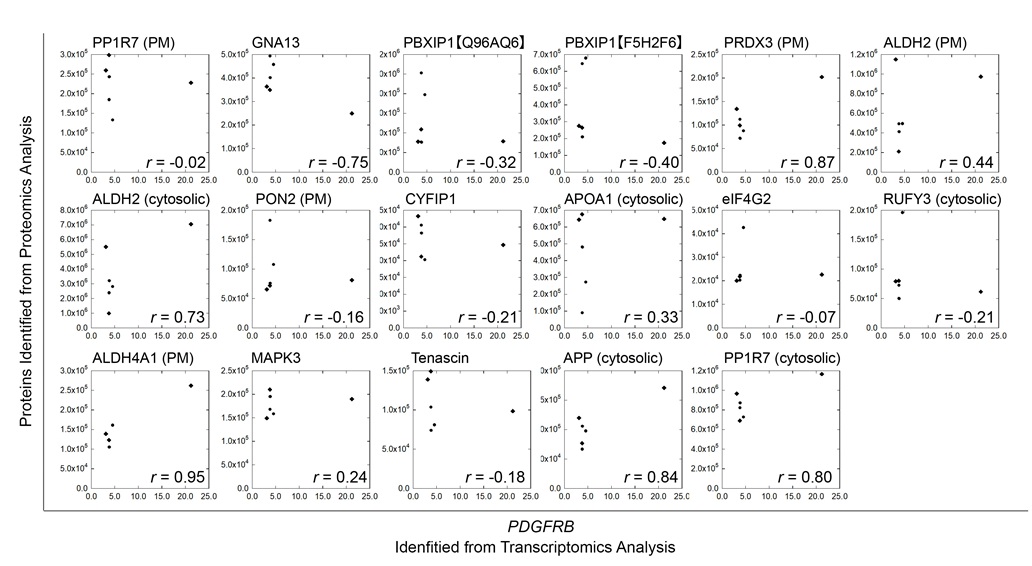
**

**H**


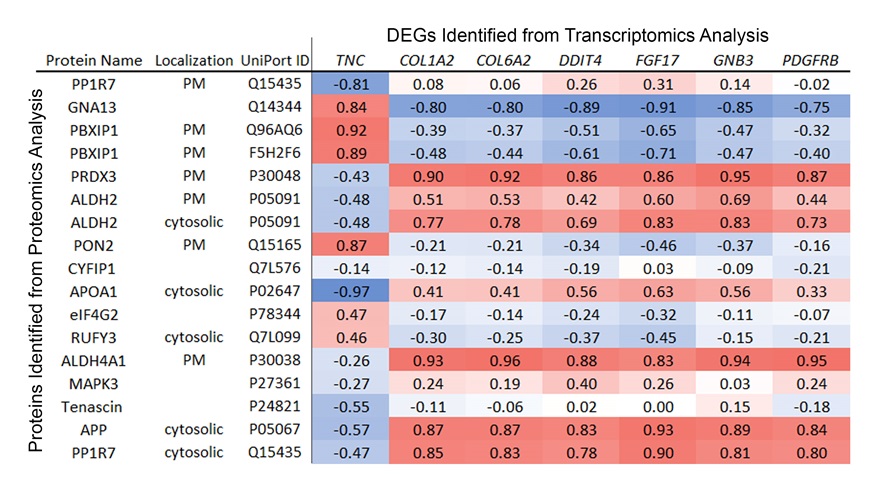


**Supplementary Figure 2.** Relationships between the transcriptomics and the proteomics factors

(**A-G**) The amounts of the identified 17 proteins were plotted against the RPKM of the 7 DEGs. Diamonds: samples from schizophrenia patients. Circles: samples from control subjects.

(**H**) Heatmap of the correlation coefficients between the factors identified from the transcriptomics and the proteomics analyses. The red color suggests a positive correlation, while the blue color suggests a negative correlation.


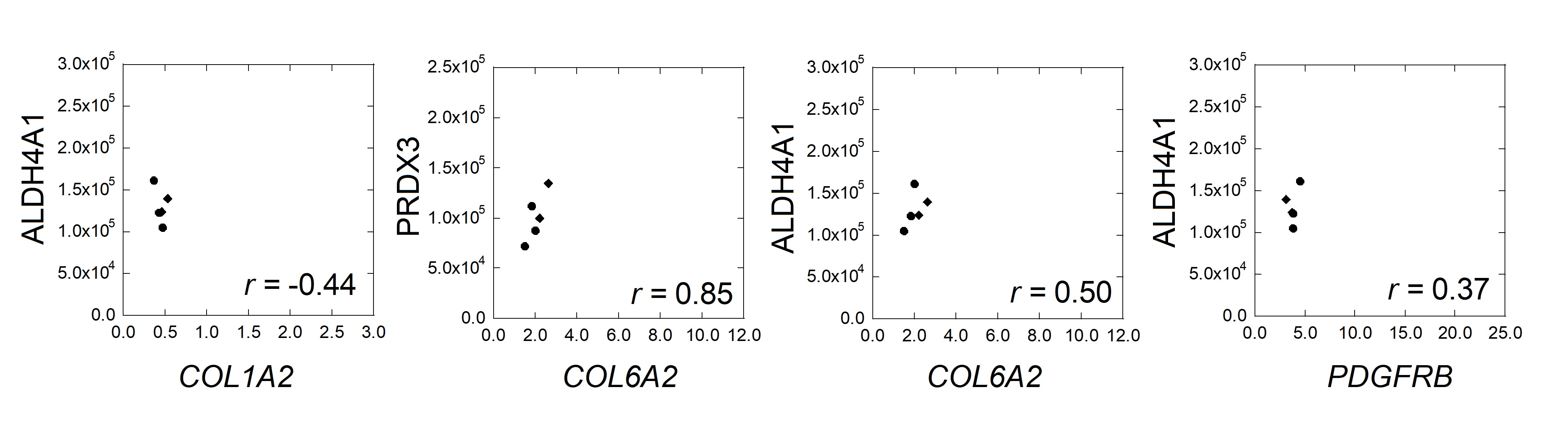


**Supplementary Figure 3.** Outlier analyses of the relationships between the transcriptomics and the proteomics factors.

Outliers were confirmed by Dixon's *Q* test. *R'/R* values of *COL1A2, COL6A2* and *PDGFRB* was 0.93, 0.88 and 0.92, respectively, all of which were greater than *Q*_0.99_(*n* = 6) = 0.74, suggesting that these points were outliers. On the other hand, *R'/R* values of *TNC* (*R'/R* = 0.15) was smaller than *Q*_0.90_(*n* = 6) = 0.56; therefore, there were no outliers in this DEG. Any of proteins PRDX3, ALDH4A1, PBXIP1 or APOA1 has no outliers of *Q*_0.99_, either. After eliminating these outliers, the correlation coefficients between *COL1A2* and ALDH4A1 was ***r***=-0.44, *COL6A2* and PRDX3 was ***r***=0.85, *COL6A2* and ALDH4A1 was ***r***=0.50, and *PDGFRB* and ALDH4A1 was ***r***=0.37. All these correlation coefficients did not exceed the α level of 0.01 (|***r***|>0.917).

**A**


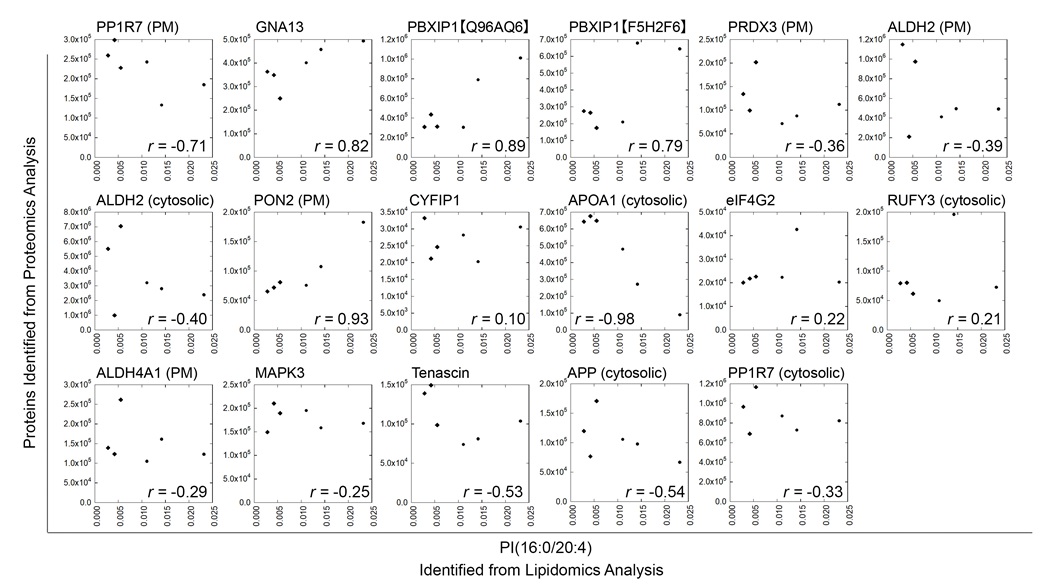


B


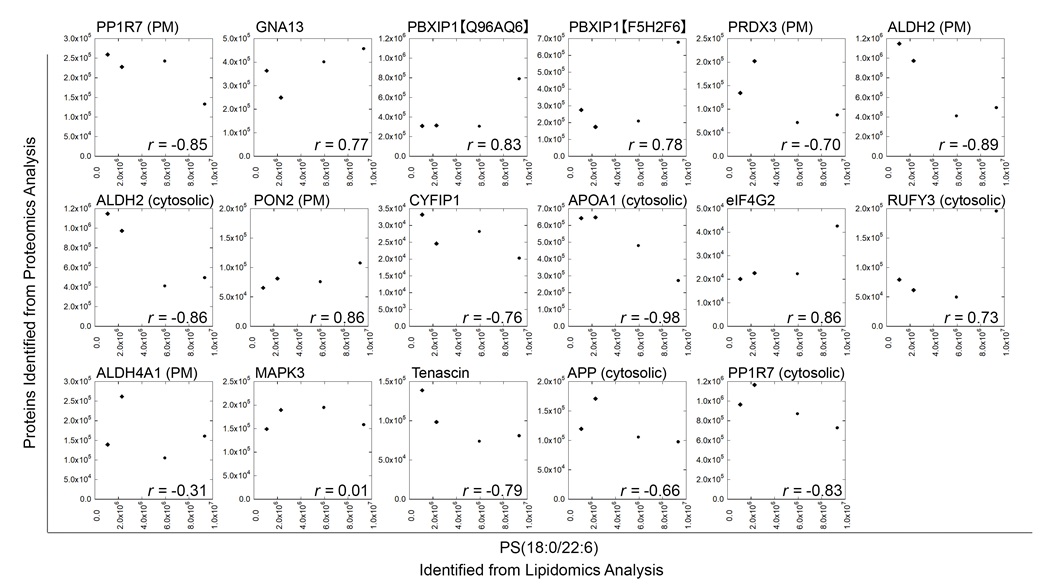


C


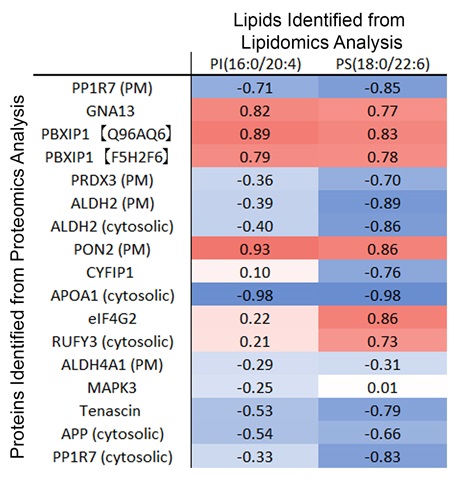


**Supplementary Figure 4.** Relationships between the lipidomics and the proteomics factors

(**A-B**) The signal intensity of the lipids, PI(16:0/20:4) and PS(18:0/22:6) were plotted against the amounts of the identified 17 proteins. Diamonds: samples from schizophrenia patients. Circles: samples from control subjects.

(**C**) Heatmap of the correlation coefficients between the factors identified from the lipidomics and the proteomics analyses. The red color suggests a positive correlation, while the blue color suggests a negative correlation.
